# Spondylodiscite granulomateuse: surtout la tuberculose mais ne pas omettre le lymphome

**DOI:** 10.11604/pamj.2016.25.98.3649

**Published:** 2016-10-19

**Authors:** Ali Zinebi, Adil Rkiouak, Yousef Akhouad, Ahmed Reggad, Zohour Kasmy, Mostafa Boudlal, Abdelhamid Nait Lho, Moncef Rabhi, Mohamed Sinaa, Khalid Ennibi, Jilali Chaari

**Affiliations:** 1 Service de Médecine A HMIM V, Rabat, Maroc; 2 Service d'Anatomopathologie HMIM V, Rabat, Maroc

**Keywords:** Lymphome vertébral, spondylodiscite tuberculose, ostéite granulomateuse, Vertebral lymphoma, tuberculous spondylodiscitis, granulomatous osteitis

## Abstract

Les douleurs lombaires relèvent d'étiologies multiples dont le diagnostic peut être source de grandes difficultés. Le lymphome rachidien primitif est rare et son diagnostic nécessite une biopsie souvent scanoguidée. Un homme de 30 ans, était hospitalisé pour lombalgies inflammatoires évoluant dans un contexte d'altération de l'état général avec à l'examen des douleurs à la palpation des apophyses épineux L2L3, sans syndrome tumoral périphérique. Le bilan biologique montrait un syndrome inflammatoire. Le bilan morphologique était en faveur d'une spondylodiscite. La première biopsie montrait une ostéite granulomateuse. L'aggravation clinique et radiologique sous anti bacillaire a mené à reconsidérer le diagnostic et la deuxième biopsie confirme le diagnostic du lymphome. Le diagnostic de tuberculose osseuse en particulier vertébrale nécessite une confirmation bactériologique et ou histologique pour ne pas méconnaître un lymphome osseux primitif.

## Abstract (en)

Lower back pain is due to multiple etiologies that make diagnosis difficult. Primitive spinal lymphoma is rare and its diagnosis often requires ultrasound-guided biopsy. A 30-year old man hospitalized for inflammatory lumbago evolving within the context of an impaired general condition. Phisical examination revealed pain on palpation of the L2-L3 vertebral apophysis without peripheral tumor syndrome. Laboratory tests showed an inflammatory syndrome. Morphological assessment was in favour of a spondylodiscitis. The first biopsy showed granulomatous osteitis.Clinical and radiological worsening during antibacillary treatment led to reconsider the original diagnosis and a second biopsy confirmed the diagnosis of lymphoma. The diagnosis of skeletal tuberculosis in particular spinal tuberculosis requires bacteriological or histological confirmation in order not to overlook a primitive bone lymphoma.

## Introduction

Des douleurs rachidiennes sans notion de traumatisme ou maladie disco-vertébrale connue peuvent relevées de plusieurs étiologies ; avec en particulier les localisations néoplasiques primitives ou secondaires et les infections. Dans les cas où des localisations osseuses de lymphomes non hodgkiniens (LNH) existent sans autre localisation ganglionnaire ou viscérale, le terme de « lymphome osseux primitif », anciennement « réticulosarcome » ou « lymphome de Parker et Jackson » est utilisé. Son diagnostic repose sur l’examen histologique alors que les données radiologiques prêtent confusion notamment avec la tuberculose surtout si la biopsie retrouve une lésion granulomateuse. C’est le cas de l’observation que nous rapportons.

## Patient et observation

Un jeune patient de 30 ans, se présente pour des lombalgies inflammatoires. Il ne présentait pas d’antécédents particuliers. Il se plaignait depuis 6 mois de lombalgies inflammatoires sans facteur déclanchant, mal calmées par les antalgiques usuels et les anti-inflammatoires non stéroïdiens, évoluant dans un contexte de fièvre avec sueur surtout nocturne et amaigrissement chiffré à 6 kilogrammes. L’examen clinique à l’admission retrouve un patient en assez bon état général, fébrile à 39°C, une raideur lombaire avec une distance doigt sol de 20 cm, des douleurs à la palpation des apophyses épineuses lombaires surtout en regard de L2-L3. Le reste de l’examen somatique ne retrouvait pas d’anomalie notamment à l’examen ostéo-articulaire, les aires ganglionnaires qui sont libres. Il n’y avait pas d’hépato splénomégalie.

**Figure 1:**
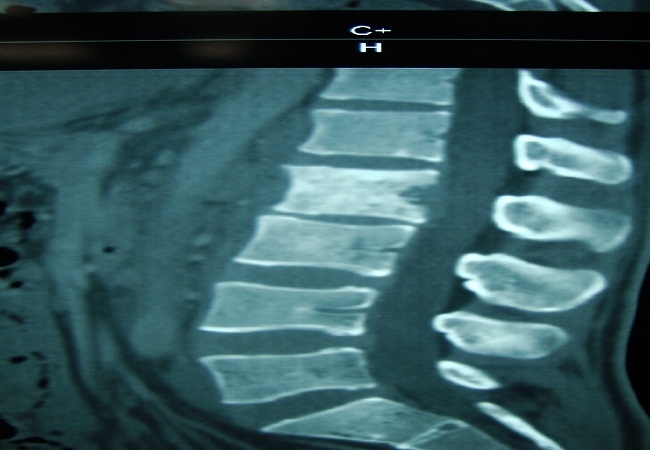
TDM du rachis montrant un processus lésionnel centré sur le corps vertébral de L2 avec présence de multiples érosions

Le bilan biologique retrouve un syndrome inflammatoire avec une CRP à 120 mg/l, une anémie inflammatoire. a 2 microglobuline était à 1.5 X la normale et LDH était normale. Le bilan morphologique ne retrouvait pas d’anomalie à la radiographie standard. La TDM du rachis lombaire révélait un processus lésionnel centré sur le corps vertébral de L2 avec présence de multiples érosions (Figure 1).

**Figure 2:**
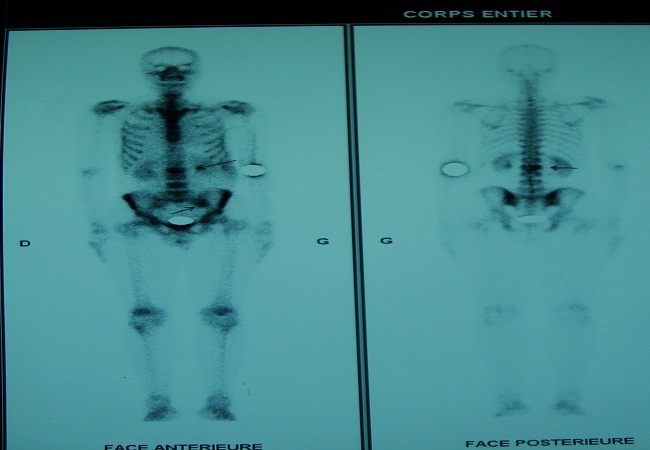
Scintigraphie osseuse montrant une fixation normale et symétrique du radiotraceur hormis deux hyperfixations au niveau de L2 et le versant iliaque de l’articulation sacro-iliaque gauche

L’IRM du rachis montrait une anomalie de signal du corps vertébral au niveau L2 en hypo signal en T>1 et hyper signal en T 2. La scintigraphie osseuse au Tc99m-HMDP retrouve deux hyperfixations au niveau de L2 et le versant iliaque de l’articulation sacro-iliaque gauche (Figure 2). Le complément IRM du bassin ne retrouve pas d’anomalie. La biopsie scano-guidée de L2 retrouve une ostéite granulomateuse. La TDM thoracoabdominale ne montrait pas d’anomalie en particulier pas de lésion du parenchyme pulmonaire. Le patient a été mis sous anti bacillaire malgré la négativité du bilan phtysiologique vu le caractère endémique de la tuberculose au Maroc, les signes généraux, l’aspect radiologique et les données histologiques.

**Figure 3:**
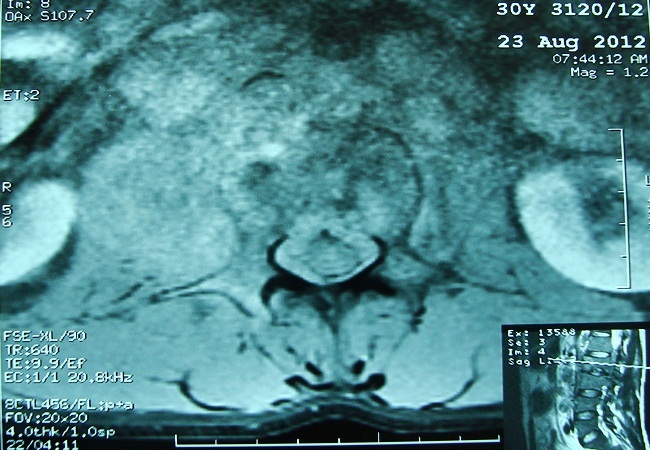
IRM du rachis trouvant anomalie de signal au niveau de L2 en hypersignal STIR et hyposignal T1 se rehaussant après injection du produit de contraste avec épaississement des parties molles en regard du muscle psoas droit

**Figure 4:**
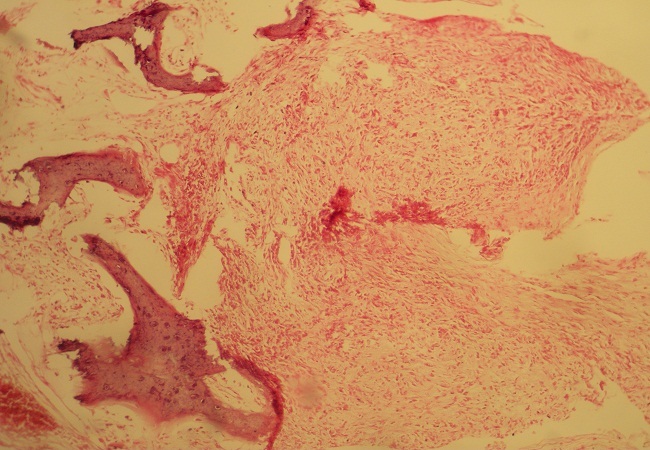
Aspect au grossissement X 100, montrant prolifération lymphomateuse avec atteinte osseuse (HEx100)

**Figure 5:**
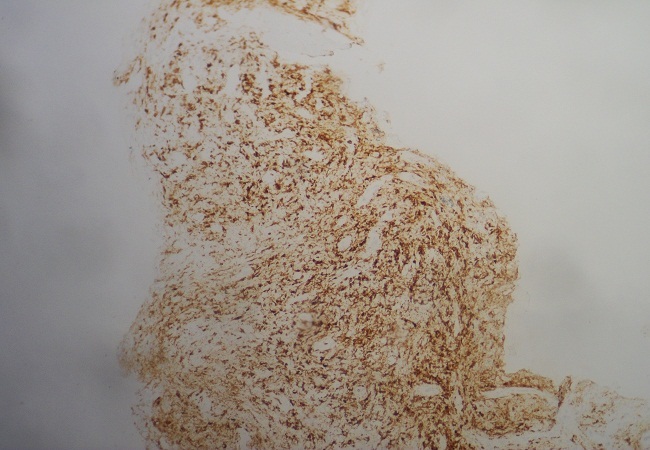
Immunomarquage des cellules tumorales par le CD 20 (HEx100)

L’évolution est marquée par l’accentuation de la douleur devenant permanente malgré le traitement par les antalgiques de troisième palier. Le contrôle radiologique retrouvait à l’IRM rachidienne un processus lésionnel centré sur le corps vertébral de L2 avec présence de multiples érosions associé à une extension épidurale et un épaississement hétérogène du muscle psoas sans collection nettement visible (Figure 3). Une seconde biopsie vertébrale montrait un aspect de lymphome malin non hodgkinien (Figure 4, Figure 5)

## Discussion

Les manifestations osseuses surviennent le plus souvent au cours d’un lymphome connu, mais dans environ 25 % des cas, elles sont présentes lors du diagnostic de la maladie, voire sont révélatrices. Les lymphomes osseux primitifs représentent 38 % des lymphomes avec atteinte osseuse et 3 % de l’ensemble des tumeurs osseuses malignes [1].

Les localisations osseuses au cours des lymphomes surviennent dans des zones de moelle osseuse active, rouge, avec une atteinte métaphysaire préférentielle des os longs. Les localisations osseuses prédominent aux membres, et surtout au fémur, atteint dans 20 % des cas ; le rachis, surtout dorsal et lombaire, et le bassin, surtout l’aile iliaque. Sont également des sites de prédilection, atteints dans 20 % des cas pour chacune de ces localisations; le squelette thoracique est affecté dans 15 % des cas, et le crâne dans 10 % des cas [1, 2]. Le lymphome isolé du plateau vertébral est très rare [3]. Il touche une population jeune de la deuxième à la quatrième décennie de vie. L’atteinte osseuse n’est pas synonyme de l’atteinte médullaire du lymphome qui est présente dans plus de 50 % des LNH à présentation ganglionnaire.

Les compressions médullaires dues à des tumeurs solides constituent 5 p. 100 des cas dont 15 p100 sont des lymphomes malins non hodgkiniens. La responsabilité du lymphome Hodgkinien est estimée à 5 p. 100 et concerne alors une maladie évoluée à extension ganglionnaire et viscérale diffuse [4]. Quant aux localisations primitivement rachidiennes, en l’absence de toute atteinte ganglionnaire, elles sont très rares et seules quelques observations ont été rapportées dans la littérature. Un début extraganglionnaire de la maladie de Hodgkin surviendrait dans moins de 0.25 p. 100 des cas [5]. Quand l’infiltration lymphomateuse est confinée à la cavité médullaire, la destruction osseuse peut être insuffisante pour donner des lésions radiologiques comme c’est le cas de notre observation, et la scintigraphie osseuse est d’un grand intérêt dans ces cas, mettant en évidence une hyperfixation. Dans moins de 5 % des cas, la scintigraphie osseuse peut faire défaut, surtout dans les formes lytiques. L’imagerie par résonance magnétique (IRM) est également plus sensible que les radiographies, permettant de mettre en évidence des lésions ostéomédullaires parfois non radiologiquement décelables. L’infiltration lymphomateuse apparaît en hyposignal ou donne un signal hétérogène en T1, avec une augmentation du signal après injection de gadolinium, et un hypersignal en T2. Dans les régions riches en moelle osseuse, les séquences comportant une suppression des graisses (STIR) augmentent la spécificité de la technique, les zones tumorales apparaissant en hypersignal, contrastant avec l’hyposignal de la moelle normale où la graisse est effacée [6].

Notre observation à la particularité d’être révélée par une lésion simulant un mal de Pott avec une spondylodiscite, une infiltration du muscle psoas avec à la première biopsie une lésion granulomateuse. Dans la littérature, des cas d’association entre une hémopathie en particulier un lymphome et une tuberculose ont été rapportés ; et une relation de cause à effet a été suggérée.

La survenue d’une tuberculose, quel que soit son site, lors du traitement d’une hémopathie, n’est pas rare, souvent par réactivation. Cependant, la survenue d’un lymphome semble pouvoir aussi être attribuée à une inflammation chronique précessive, comme lors d’une tuberculose non traitée. En effet, l’inflammation et la prolifération lymphocytaire médullaire liées à la tuberculose auraient pu favoriser la transformation des lymphocytes en lymphocytes tumoraux CD5+ CD20+ CD23+. A l’inverse, la survenue d’une tuberculose favorisée par l’immunodépression aurait pu être secondaire au développement d’un clone B tumoral [7, 8]. Malgré ces associations décrites, la tuberculose n’a pas été retenue, dans notre cas, comme diagnostic finale en raison de l’aggravation de la symptomatologie et des lésions radiologiques sous traitement anti bacillaire.

Le diagnostic du lymphome vertébral doit être évoqué devant une atteinte vertébrale orientatrice surtout si la biopsie ne retrouve pas de caséum et l’étude bactériologique avec culture ne montre pas de Bacille de Koch. L’étude cytologique n’est pas suffisante et doit être complétée par une biopsie généralement scano-guidée [8–10].

Le pronostic et l’attitude thérapeutique au cours des hémopathies malignes sont dictés par le type histologique de la prolifération tumorale et l’extension de la maladie. Le pronostic des lymphomes osseux est plus favorable que celui des autres tumeurs malignes osseuses, avec un taux de survie à cinq ans de 40-50% [3]. Le pronostic des formes localisées (stade IE) est meilleur que dans les formes à localisations osseuses multiples (stade IV). Enfin, la survenue d’une atteinte osseuse au cours d’une hémopathie maligne serait le témoin d’une évolutivité et d’un mauvais pronostic de la maladie.

## Conclusion

Le lymphome rachidien reste rare et de pronostic péjoratif surtout quand elle s’accompagne d’atteinte endocavitaire exposant au risque de compression médullaire. La TDM et surtout l’IRM rachidienne reste l’examen radiologique de référence dans le diagnostic topographique et dans le bilan d’extension. Cependant ; le diagnostic positif repose entièrement sur les données de l’examen anatomopathologique.
